# Activation of protease-activated receptor 2 reduces glioblastoma cell apoptosis

**DOI:** 10.1186/1423-0127-21-25

**Published:** 2014-03-26

**Authors:** Ran Luo, Xiongwei Wang, Yuanxun Dong, Lei Wang, Chunlei Tian

**Affiliations:** 1 Department of Neurosurgery, Institute of Neurosurgery, Yichang Central People’s Hospital & The First Clinical Medical College of Three Gorges University, Yichang, Hubei 443003, P.R. China

**Keywords:** Glioma, Tryptase, Protease-activated receptor 2, Signal transducer and activator of transcription 3, p53

## Abstract

**Background:**

The pathogenesis of glioma is unclear. The disturbance of the apoptosis process plays a critical role in glioma growth. Factors regulating the apoptosis process are to be further understood. This study aims to investigate the role of protease activated receptor-2 (PAR2) in regulation the apoptosis process in glioma cells.

**Results:**

The results showed that U87 cells and human glioma tissue expressed PAR2. Exposure to tryptase, or the PAR2 active peptide, increased STAT3 phosphorylation in the radiated U87 cells, reduced U87 cell apoptosis, suppressed the expression of p53 in U87 cells.

**Conclusions:**

Activation of PAR2 can reduce the radiated U87 cell apoptosis via modulating the expression of p53. The results implicate that PAR2 may be a novel therapeutic target in the treatment of glioma.

## Background

Malignant gliomas are the most common and aggressive adult brain tumors including anaplastic glioma and glioblastoma multiforme; the clinical outcomes are very poor; it is associated with a life expectancy of less than two years in general. The pathogenesis of glioma is unclear. Thus, it is urgent to understand the pathogenesis and find novel therapeutic targets for the treatment of glioma [1,2].

Apoptosis is a process of programed cell death. Unlike necrosis, apoptosis produces cell fragments called apoptotic bodies that phagocytic cells are able to engulf and quickly removed before the contents of the cell can spill out onto surrounding cells and cause damage [3]. Under physiological conditions, apoptosis is tightly regulated by a number of factors, such as the signal transducer and activator of transcription 3 (STAT3), which inhibits apoptosis via suppressing p53 expression; the latter is a tumor suppressor protein [4]. In addition to its importance as a biological phenomenon, defective apoptotic processes have been implicated in an extensive variety of diseases. Excessive apoptosis causes atrophy, destroys functional cells, such as damaging islet to induce diabetes [5], whereas an insufficient amount of apoptosis results in uncontrolled cell growth, such as cancer [6]; the initiation mechanism remains to be further investigated.

Protease-activated receptor (PAR) 2 is a member of the large family of 7-transmembrane receptors that couple to guanosine-nucleotide-binding proteins. It is activated by trypsin. PAR2 can be activated by proteolytic cleavage of its extracellular amino terminus. The new amino terminus functions as a tethered ligand and activates the receptor [7]. Recent reports suggest that PAR2 is involved in the regulation of apoptosis [8]. Since defective of apoptosis plays a crucial role in the pathogenesis of cancer, we hypothesized that PAR2 might be involved in the regulation of glioma cell apoptosis. To test the hypothesis, using a glioma cell line, the U87 cells, as a study model, we observed that U87 cells expressed PAR2. Tryptase activated PAR2 to reduce U87 cell apoptosis by suppressing STAT3 phosphorylation and regulating the levels of p53 in U87 cells.

## Methods

### Reagents

Antibodies of PAR2, STAT3, p53, and a STAT3 shRNA kit were purchased from Santa Cruz Biotech (Shanghai, China). Reagents for quantitative real time RT-PCR (qRT-PCR) and Western blotting were purchased from Invitrogen (Shanghai, China). An annexin V kit and tryptase were purchased from Sigma Aldrich (Shanghai, China). The active PAR2 peptide and control peptide were purchased from Alibaba Biotech (Hangzhou, China).

### Cell culture

Glioma cell line, U87 cell, was purchased from ATCC. U87 cells were cultured in DMEM medium supplemented with 10% fetal bovine serum, 2 mM glutamine, 200 U penicillin/ml, 0.2 g/ml streptomycin at 37°C.

### Human glioma tissue collection

Surgically removed glioma tissue was collected from 3 patients (1 male, 2 females). The marginal normal tissue was collected using as the normal control tissue. The glioma tissue and normal tissue were proved by a pathologist. The using human tissue in the present study was approved by the Human Research Ethic Committee at Three Gorges University. An informed, written consent was obtained from each patient.

### Irradiation

U87 cells were irradiated with a medical linear accelerator (Varian Linear Accelerator models 2100C (/D), Varian Medical Systems, Palo Alto, CA, USA) at 500 cGy/min; the irradiation was continued until 8Gy as described previously [9]. Control cells were unirradiated. 6-8 h after the irradiation, the cells were processed for further experiments.

### qRT-PCR

Total RNA was extracted from U87 cells with the TRIzol reagents. cDNA was converted from the RNA with a reverse transcription kit. qPCR was performed with SYBR supermix in a MiniOptcon real time PCR system. The Ct values were analyzed using the 2ΔΔCT method. The target gene expression was normalized to the percentage of β-actin. The primers using in the present study include: PAR2, forward, tgctagcagcctctctctcc; reverse, ccagtgaggacagatgcaga (NCBI, AY336105). P53, forward, tggccatctacaagcagtca; reverse, ggtacagtcagagccaacct (NCBI, NM_001126114.2).

### Western blotting

Total proteins were extracted from the U87 cells and separated on SDS-PAGE (sodium dodecyl sulfate polyacrylamide gel electrophoresis), and transferred onto a nitrocellulose membrane; the membrane was blocked with 5% skim milk for 30 min, incubated with the first antibodies (100 ng-200 ng/ml) overnight at 4°C, and followed by incubating with the secondary antibodies for 1 h. The membranes were washed with TBST (Tris buffered saline-Triton X-100) after each incubation. The immune complexes were developed with the enhanced luminol-based chemiluminescent substrate. The results were recorded with X films.

### Flow cytometry

Apoptosis of U87 cells was assessed by flow cytometry. The live cells were stained with propidium iodide (PI) and annexin V kit. The PI^+^ cells were gated out first; the remaining cells were assessed to determine the frequency of annexin V^+^ cells.

### RNA interference (RNAi)

The STAT3 gene or PAR2 gene was knocked down in U87 cells with commercial RNAi reagent kits following the manufacturer’s instructions. The peak value of the STAT3 or PAR2 knockdown was reached in 6-8 days after the transduction.

### Statistics

The data were presented as mean ± SD. Differences between groups were determined by Student *t* test. P < 0.05 was set as a significant criterion.

## Results

### Glioma cells express PAR2

**Figure 1:**
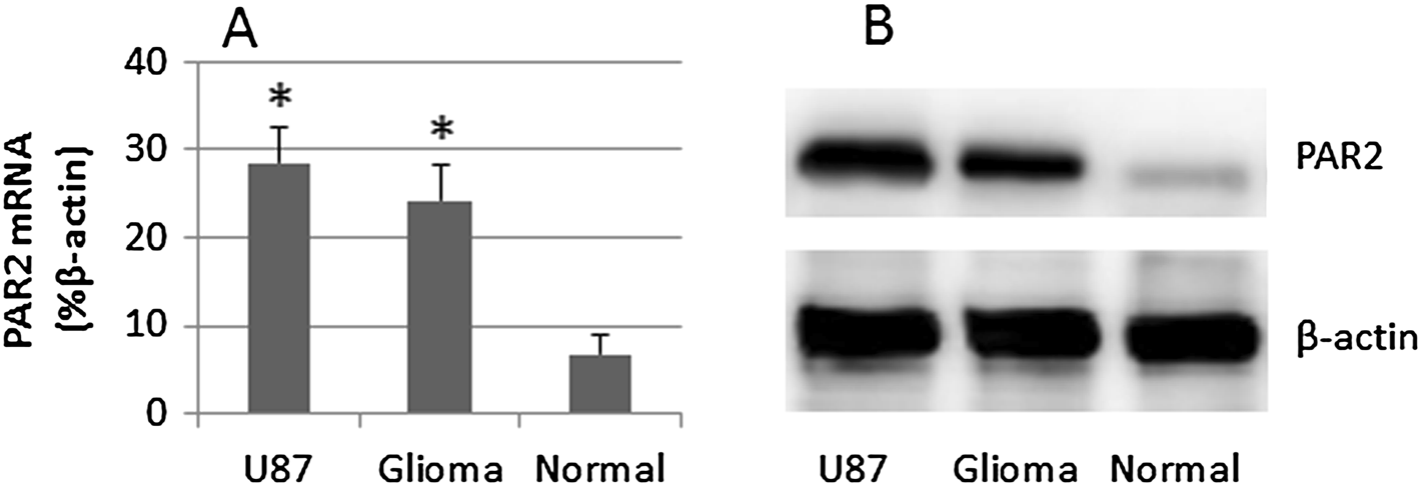
**Expression of PAR2 is increased in glioam cells.** Total RNA and proteins were extracted from surgically removed glioma tissue (3 patients), the marginal normal tissue and U87 cells; the samples were analyzed by qRT-PCR and Western blotting. **A**, the bars indicate the mRNA levels of PAR2 (mean ± SD; *, p < 0.01, compared with normal tissue). **B**, the immune blots indicate the protein levels of PAR2. The data represent 3 separate experiments.

We firstly assessed the expression of PAR2 in the glioma tissue and glioma cell line, the U87 cells. As shown by qRT-PCR and Western blotting, the expression of PAR2 was detected in U87 cells and glioma tissue at both mRNA levels and protein levels. Much less PAR2 levels were detected in the normal brain tissue (Figure 1).

### Tryptase reduces radiation-induced U87 cell apoptosis

**Figure 2:**
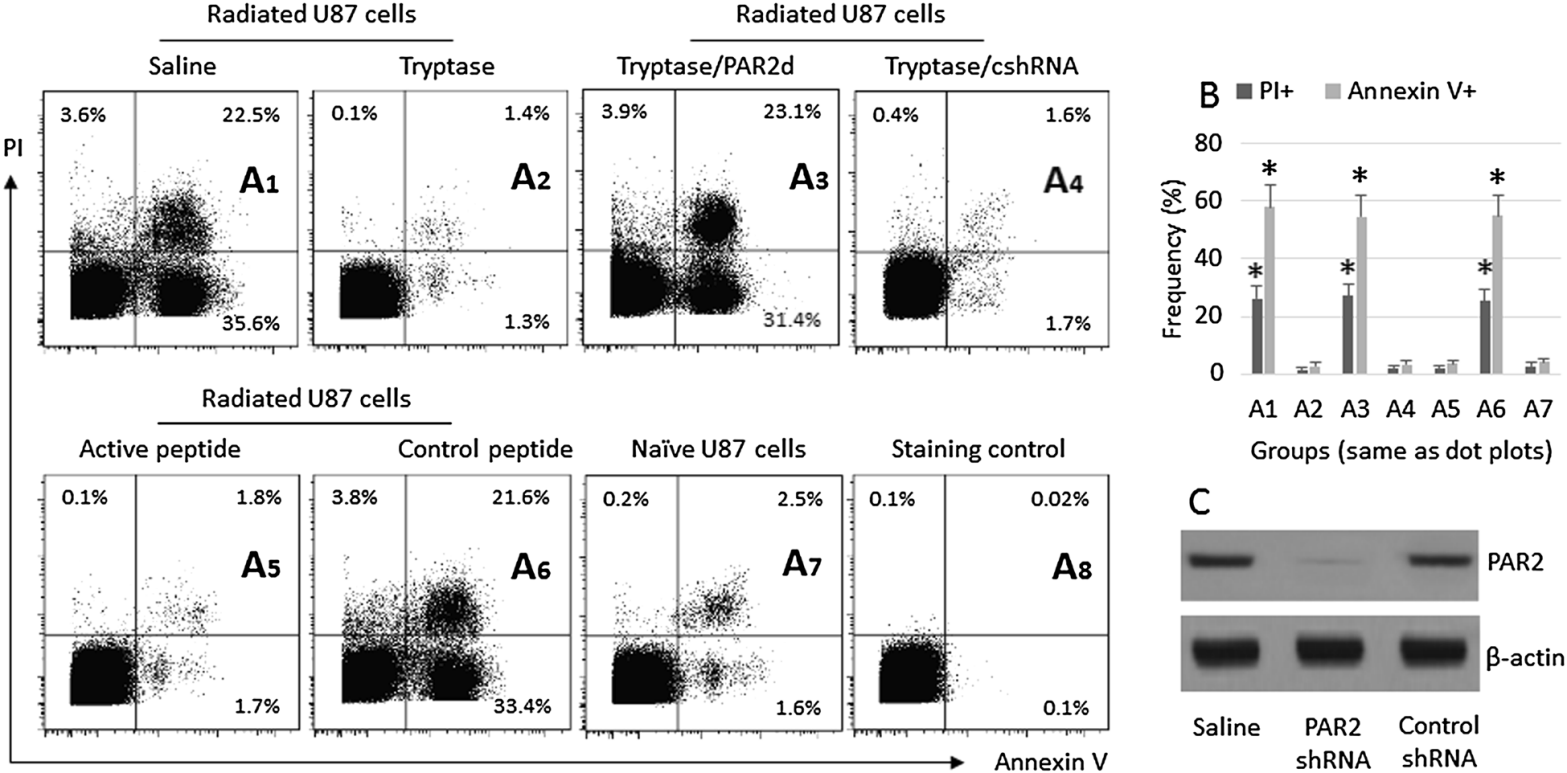
**Tryptase inhibits U87 cell apoptosis.** The treatment of U87 cells is denoted above each dot plot panel. Radiation: U87 cells were treated with radiation (8Gy) in the culture. Dose of tryptase (control peptide and active peptide): 10 μg/ml. **A**, the dot plots indicat the frequency of PI^+^ U87 cells or/and Annexin V^+^ cells, which are summarized in **B**. PAR2d: U87 cells with the PAR2 gene knockdown. cshRNA: U87 cells treated with control shRNA. **C**, the PAR2 gene knockdown results. *, p < 0.01, compared with group A7 (mean ± SD). The data are from 3 separate experiments.

Mast cells are associated with cancer growth [10]. Tryptase is one of the major chemical mediators of mast cells; it cleaves PAR2 to activate the PAR2-bearing cells. We postulate that tryptase activates U87 cells and influences the process of apoptosis induced by other factors such as radiation. Thus, we treated U87 cells with radiation in the presence or absence of tryptase or the PAR2 active peptide. As shown by flow cytometry data, about 4% apoptotic cells were detected in naïve U87 cells; after radiation, the apoptotic U87 cells reached 56%, which was abolished by the presence of tryptase or the PAR2 active peptide in the culture (Figure 2).

### Tryptase suppresses radiation-induced STAT3 phosphorylation in U87 cells

**Figure 3:**
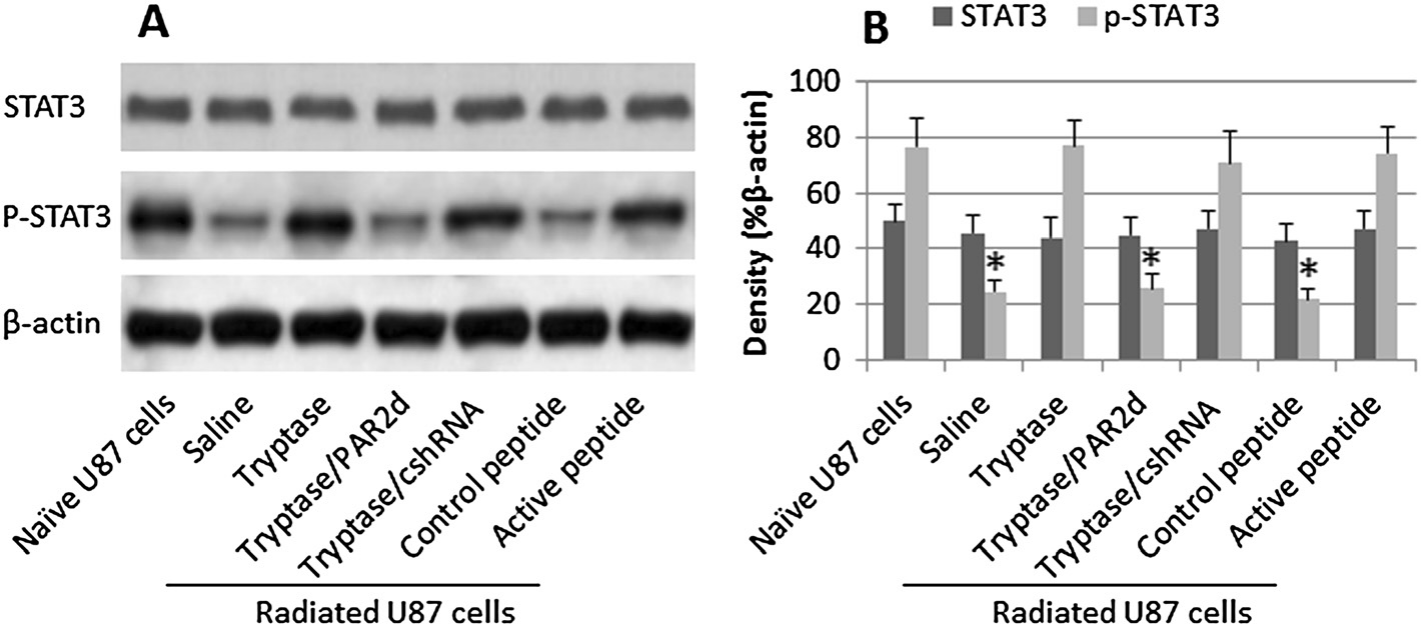
**Tryptase increases STAT3 phosphorylation in radiated U87 cells. A**, the treatment of radiated U87 cells is denoted below the immune blots. **B**, the bars indicate the blot density of panel **A**. *, p < 0.01, compared with naïve U87 cells. PAR2d: PAR2-deficient U87 cells. cshRNA: Control shRNA. The data represent 3 separate experiments.

STAT3 is involved in cancer growth [11]. Based on the data of Figures 1 and 2, we infer that STAT3 is involved in the inhibition of the radiation-induced U87 cell apoptosis in the presence of tryptase. Thus, we treated U87 cells with the same procedures of Figure 2. The results showed that the STAT3 phosphorylation was detected in naïve U87 cells, which was markedly suppressed by radiation. The treatment with tryptase or active PAR2 peptide significantly suppressed the phosphorylation of STAT3, which was abolished by silencing the PAR2 gene by RNAi (Figure 3). The results indicate that tryptase can repress the phosphorylation of STAT3 in U87 cells.

### Tryptase alters the expression of p53 in radiated U87 cells

**Figure 4:**
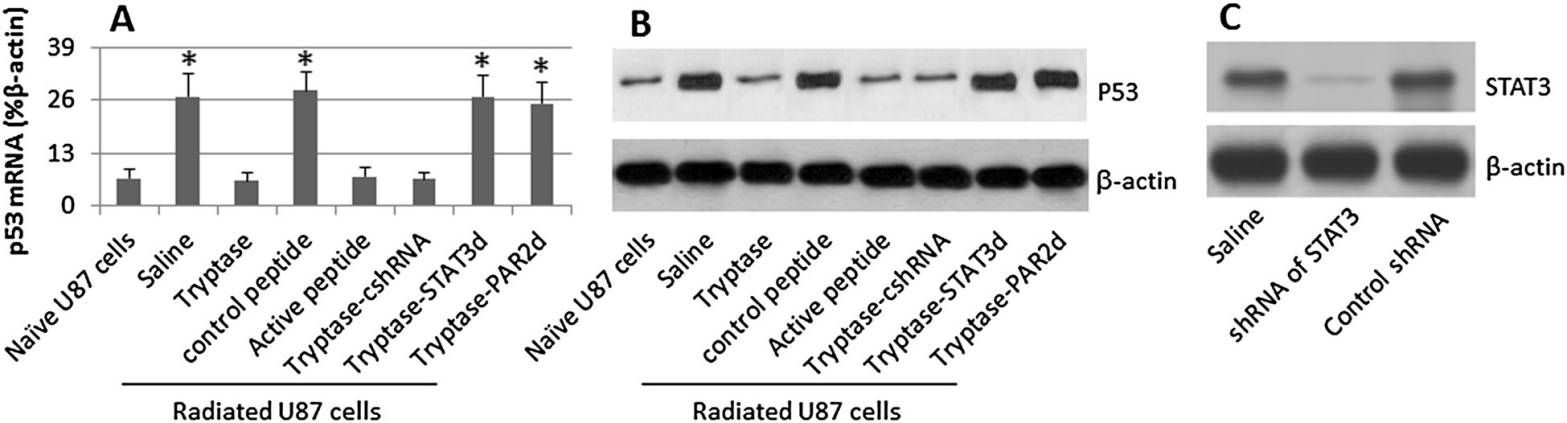
**Tryptase regulates expression of p53 in U87 cells.** STAT3-sufficient or deficient U87 cells were cultured and radiated in the presence of tryptase or active PAR2 peptides for 48 h. The cells were analyzed for the expression of p53 by qRT-PCR and Western blotting respectively. **A**, the bars indicate the mRNA levels of p53 (mean ± SD; *, p < 0.01, compared with naive group). **B**, the immune blots indicate the protein levels of p53. **C**, the STAT3 gene knockdown results. The data represent 3 separate experiments. STAT3d: STAT3 deficient U87 cells. PAR2d: PAR2-deficinet U87 cells. cshRNA: Radiated U87 cells were treated with control shRNA. The data represent 3 separate experiments.

P53 protein is an important molecule in the process of apoptosis. Whether tryptase regulates the expression of p53 in U87 cells, thus regulate the apoptosis of U87 cells, is unclear. We next assessed the levels of p53 in radiated U87 cells after stimulating by tryptase. The results showed that tryptase or the active PAR2 peptide markedly suppressed the levels of p53 in U87 cells. To further test the role of STAT3 in the tryptase-regulated p53 expression in U87 cells, a batch of U87 cells were knocked down the gene of STAT3, treated with radiation and exposed to tryptase in the culture. Indeed, the expression of p53 was un-affected, similar to the saline group or the PAR2-null U87 cells (Figure 4). The results implicate that tryptase alters the apoptosis rate in radiated U87 cells via regulating the expression of p53; STAT3 plays an important role in the process.

## Discussion and conclusions

The present study revealed that the glioma cell line, U87 cells, and human glioma tissue, expressed high levels of PAR2. Upon exposure to tryptase, the radiation-induced U87 cell apoptosis was reduced, the phosphorylation of STAT3 was increased, p53 levels in U87 cells was suppressed.

In line with published data [12], we observed that the frequency of apoptotic U87 cells was increased after irradiation. Apoptosis is a physiological phenomenon. The significance of apoptosis is to remove senescent cells [13] and the over functional cells, such as activated T cells (a phenomenon designated the activation induced cell death) [14]. Deregulation of apoptosis is associated with the pathogenesis of a number of disorders, such as tumor cell growth [15]. One of the novel findings of the present study is that the expression of PAR2 is higher in U87 cells and human glioma tissue than normal brain tissue; activation of PAR2 by exposing to tryptase or the active PAR2 peptide can suppress the radiation-induced U87 cell apoptosis. The results implicate that activation of PAR2 can dirsturb the radiation-induced U87 cell apoptosis.

Published data indicate that mast cells are residential cells in the brain [16], such as in the thalamus, entorhinal cortex, hippocampus and the leptomeninges [17-19]. Radiation stimulation is one of the factors inducing mast cell activation [20]. Upon activation, mast cells release mediators in a piecemeal fasion or anaphylactic fasion [21,22]. The released tryptase may contact the body cells to regulate the apoptotic process. Tryptase is one of the major chemical mediators of mast cells. Previous reports indicate a close association between mast cells and cancers. Ma et al suggest that dynamic mast cell-stromal cell interactions promote growth of pancreatic cancer [23]. Khan et al indicate that mast cells are associated in the pathogenesis of colitis-induced colon cancer [24]. Together with the present data, mast cell-derived tryptase may contribute to the pathogenesis of cancer by disturbing the process of apoptosis in the body cells. To approve the inference, further experiments are needed.

STAT3 is an important mediator of tumor cell survival, growth, and invasion in a large group of glioma [25]. Hu et al indicate that STAT3 has a crucial role in cervical cancer progression, metastasis and develop to anti-cancer therapies [26]. Carbajo-Pescador et al. propose that blocking STAT3 has the therapeutic potential in malignant tumors [27]. The phosphorylation of STAT3 can inhibit p53 expression, or the inhibition of STAT3 induces p53 accumulation [4]. Our data have enriched the knowledge in this axis of STAT3-p53-cancer by providing evidence that tryptase can increase the phosphorylation of STAT3, so as to suppress the expression of p53. Mast cells regularly distribute all over the body of both healthy person and patients with cancer, and can be activated to release tryptase by a large number of events, such as allergy [28], exposure to microbial products [29] and radiation [30]. The radiation resistance of cancer is a common syndrome in the treatment of cancer; the pathogenesis is not fully understood. The present data provide evidence that mast cell-derived tryptase may be involved in the radiation resistance by modulating STAT3 phosphorylation and p53 expression in cancer cells. To prove the inference, further investigation is needed.

## Competing interests

The authors declare that they have no competing interests.

## Authors’ contributions

RL, YD, LW and CT performed experiments, analyzed data and reviewed the manuscript. XW designed the project and wrote the paper. All authors read and approved the final manuscript.
